# Supplementary material for: No Increased Risk of Major Adverse Cardiovascular Events following Nicotinamide Exposure
Source: medRxiv. 2024 Sep 16:2024.09.16.24313743. Preprint. [Version 1] doi: 10.1101/2024.09.16.24313743 (PMC11451707; doi:10.1101/2024.09.16.24313743)

Supplemental figure 1. Standardized Mean Difference plot showing the mean differences between exposed patients and the pool of unexposed patients in variables before (white circles) and after (black circles) propensity score matching. The dotted vertical line is 0.05 and the vertical solid line is at 0.1


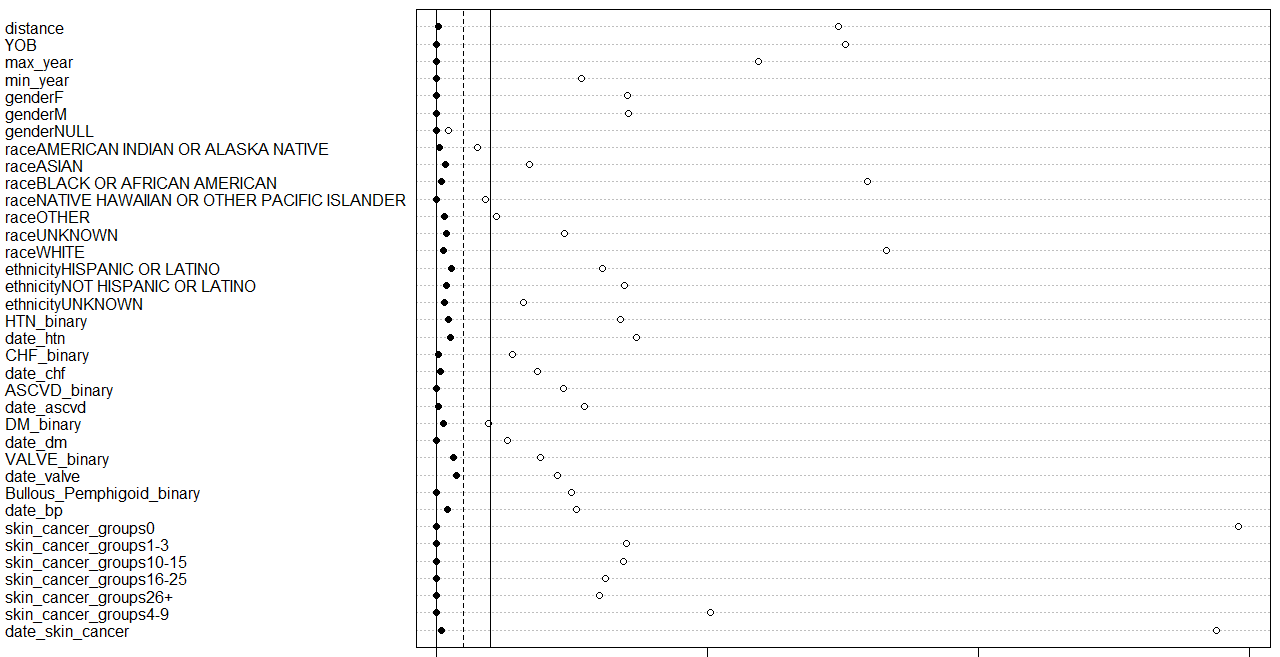

Supplement: Supplement 1 [file media-1.docx]
